# Malnutrition Risk and Depressive Symptoms in Community-Dwelling Older Adults

**DOI:** 10.3390/nu18213478

**Published:** 2026-10-23

**Authors:** Radmila Matijevic, Milan Milinkov, Stefan Janicic, Srdjan Sarac, Nikola Slokar, Dora Selesi

**Affiliations:** 1 Faculty of Medicine in Novi Sad, University of Novi Sad, 21000 Novi Sad, Serbia; milan.milinkov@mf.uns.ac.rs (M.M.); srdjan.sarac@mf.uns.ac.rs (S.S.); 2 University Clinical Centre of Vojvodina, 21000 Novi Sad, Serbia; stefan.jancic@kcv.rs (S.J.); nikola.slokar@kcv.rs (N.S.); 3 Faculty of Sciences, University of Novi Sad, 21000 Novi Sad, Serbia; dora@dmi.uns.ac.rs

**Keywords:** malnutrition, Mini Nutritional Assessment, older adults, depression, visceral adipose tissue

## Abstract

Objectives: To describe nutritional status in a volunteer cohort of older adults attending community sarcopenia screening and to explore factors associated with risk of malnutrition, with secondary interest in whether nutritional risk coincides with, rather than excludes, higher adiposity. Design: Cross-sectional, single-centre, exploratory analysis. Setting: A community sarcopenia-screening programme in Vojvodina, Serbia, open to volunteers recruited through pensioners’ associations. Participants: Self-selected, community-dwelling volunteers aged 60 years and older. The analytic sample comprised 388 participants with a complete Mini Nutritional Assessment (MNA). Measurements: Nutritional status was assessed with the full MNA. Depressive symptoms (GDS-15), cognition (MoCA), education, handgrip strength, gait speed, and body composition, including visceral adipose tissue (VAT) by dual-energy X-ray absorptiometry, were recorded. The primary analysis was a multivariable logistic regression for risk of malnutrition (at-risk versus normal) using predictors assessed separately from the MNA; the continuous MNA score and body composition were secondary or exploratory. Results: No participant met the criterion for established malnutrition; 52 of 388 (13.4%, 95% CI 10.2 to 17.2) were at risk of malnutrition. Depressive symptoms were the dominant and most robust correlate, in both the binary model (odds ratio 1.29 per GDS-15 point, 95% CI 1.18 to 1.41) and the continuous-score model. Because the MNA includes a depression/dementia item, this association may partly reflect construct overlap and should not be interpreted as wholly external to the nutritional assessment. Lower education was suggestive in the binary model (odds ratio 2.08, 95% CI 0.95 to 4.56) and significant only for the continuous score. Nutritional risk was not concentrated in the leanest participants: after adjustment for age and sex, higher visceral adipose tissue was associated with at-risk status (odds ratio 1.46 per kg, 95% CI 1.02 to 2.10), but this attenuated after adjustment for depressive symptoms and showed no formal effect modification by sex. Comorbidity and marital status were not associated with nutritional risk. Conclusions: In this volunteer, relatively preserved cohort, risk of malnutrition was most strongly associated with depressive symptoms, although the MNA itself includes a depression/dementia item, with a weaker educational gradient, and was not a condition of low body mass; the relationship with visceral adiposity was exploratory and non-independent. These cross-sectional associations support nutritional screening coupled with evaluation of mood, and caution against excluding older adults with normal or high body mass.

## 1. Introduction

Malnutrition in later life is common, under-recognised and consequential. Among community-dwelling older adults, it is associated with loss of independence, impaired physical function, more frequent hospital admission and higher mortality [1]. Because overt undernutrition is often preceded by a longer phase of nutritional risk, screening tools that detect this earlier phase are central to preventive geriatric care.

The Mini Nutritional Assessment (MNA) is among the most widely used instruments for this purpose [2,3]. It integrates anthropometric, dietary, global and self-perception items into a single score and classifies older adults as well nourished, at risk of malnutrition, or malnourished [2,3,4,5,6]. Prevalence estimates vary markedly by setting, being highest in hospital and institutional samples and lowest in community populations, which typically comprise more functionally intact and mobile individuals [3,7].

The determinants of nutritional risk are multifactorial and extend well beyond food intake. Depressive symptoms, cognitive impairment, social circumstances such as education and living arrangements, polypharmacy and reduced physical performance have all been linked with poorer nutritional status [8,9,10,11]. Which of these dominate in any given population depends on that population’s characteristics, and evidence from community cohorts in South-Eastern Europe remains limited.

A further consideration is that nutritional risk and adiposity are frequently assumed to be inversely related, so that a normal or high body mass is taken to exclude nutritional concern. This assumption is increasingly questioned. Older adults may carry substantial fat mass, including visceral adipose tissue, while nonetheless being at nutritional risk, a pattern relevant to the wider construct of sarcopenic obesity [12]. Whether nutritional risk is confined to the leanest members of a community cohort, or coincides with higher adiposity, has practical implications for whom clinicians choose to screen.

Using data from a community sarcopenia-screening programme, we set out to describe the nutritional status of older adults and to identify the factors associated with risk of malnutrition. We placed particular emphasis on distinguishing correlates assessed separately from the MNA from variables embedded within the instrument, and on examining whether nutritional risk coincides with higher visceral adiposity rather than with leanness. All analyses are cross-sectional, associational and exploratory; the cohort was a self-selected group of volunteers, and no analysis plan was pre-registered, so the findings require confirmation.

## 2. Methods

### 2.1. Study Design and Setting

This was a cross-sectional, single-centre, exploratory analysis of participants attending a community sarcopenia-screening programme in Vojvodina, Serbia. All interested members of local pensioners’ associations were invited to attend, so the cohort is a self-selected, volunteer sample rather than a population-based one. The study is reported in accordance with the STROBE guidance for cross-sectional studies [13]. The same source cohort underlies related reports on driving and on osteosarcopenia. The University Clinical Centre of Vojvodina Ethics Committee approved the scientific analysis and publication of data collected within the community health-screening programme (No 6 Broj 00-73/7).

### 2.2. Participants

Eligible participants were community-dwelling adults aged 60 years or older who attended screening in person; all were ambulatory volunteers. Two members of the source cohort were recorded as age 59 but were within two months of their 60th birthdays and were retained; one had a complete MNA and entered the analytic cohort. From a source cohort of 699 individuals, the analytic sample comprised the 388 participants who had a complete full MNA (the core analytic sample). The remaining participants lacked a complete full MNA primarily because the assessment was not completed during the community screening visit owing to session time or logistical constraints, or because the form was incomplete; this represented operational missingness rather than a protocol-based exclusion based on nutritional status. They were therefore not eligible for the primary analysis. Because participation was voluntary, the sample is likely to over-represent healthier, more functionally intact older adults; a comparison of participants with and without a complete MNA is provided in Supplementary Table S1.

### 2.3. Nutritional Status

Nutritional status was assessed with the full Mini Nutritional Assessment [2,3], scored from 0 to 30, and participants were classified as normal (24 or above), at risk of malnutrition (17 to 23.5) or malnourished (below 17). Risk of malnutrition, defined as the combined at-risk and malnourished categories versus normal, was the primary outcome. Because no participant met the criterion for established malnutrition, this reduced in practice to a contrast of at-risk versus normal. The continuous MNA score was analysed as a secondary outcome. The full MNA was administered using the official Bosnian long-form MNA provided by the instrument distributor, Bosnian being closely related to and largely mutually intelligible with Serbian. A separately translated and validated Serbian-language version of the full MNA is not distributed, and the absence of a formally validated Serbian MNA is acknowledged as a formal limitation.

### 2.4. Other Measurements

All other instruments were administered in Serbian; the GDS-15 was used in its validated Serbian version [14,15]. Depressive symptoms were measured with the 15-item Geriatric Depression Scale (GDS-15) [14,15] and cognition with the Montreal Cognitive Assessment (MoCA; score range 0–30, with scores <26 indicating possible cognitive impairment) [16]. Education was recorded on a six-level ordinal scale and collapsed for analysis into three categories: lower (up to and including completed primary school), secondary (completed secondary school, the reference category) and higher (college, master’s or doctoral level). Handgrip strength was measured with a Jamar hydraulic dynamometer in the dominant hand, in a seated position, recording the best of three trials. Physical performance was assessed with gait speed measured over an 8-metre course and the Short Physical Performance Battery (SPPB) [17], and self-reported sarcopenia risk with the SARC-F questionnaire [18]. Body composition, including whole-body fat mass, whole-body lean mass, appendicular lean mass indexed to height squared following the EWGSOP2 framework [19], and visceral adipose tissue (VAT), was measured by dual-energy X-ray absorptiometry (GE Lunar iDXA, GE Healthcare). A fasting venous sample provided serum 25-hydroxyvitamin D, parathyroid hormone, a full lipid panel (total, HDL and LDL cholesterol, triglycerides, non-HDL cholesterol and the atherogenic index), fasting glucose and bone–mineral-related biochemistry. The Charlson comorbidity index [20] and marital status were available from the linked cohort dataset.

### 2.5. Data Quality and Cleaning

Several data issues were identified and addressed before analysis and are documented in a cleaning log. Before modelling, key variables were reviewed for allowable ranges, units, distributions, internal consistency and biological plausibility; variables linked across source files were also compared with the available source fields. Values without a defensible correction were set to missing rather than imputed. The affected observations, actions and related sensitivity analyses are summarised in Supplementary Table S11. First, the walk test underlying gait speed and the SPPB gait component had been administered over an 8-metre course but initially processed as though the distance were 4 m, producing implausibly slow recorded speeds. Gait speed was recalculated over the correct distance (corrected median 0.81 m/s), and the SPPB was rescored accordingly (corrected median 9). Second, two handgrip values were physically impossible, consistent with misplaced decimal separators, and were set to missing. Third, a subset of whole-body fat mass values fell below 5 kg, which is not biologically plausible for the accompanying body mass index values and indicated a scaling error introduced during dataset linkage; these values were set to missing for body-composition analyses. Fourth, 34 full MNA scores were marginally above the instrument maximum (all either 30.5 or 31.5). Anonymisation consisted solely of replacing names and surnames with fixed participant ID numbers; model variables were not numerically perturbed. The excess MNA was 0.5 or 1.5 points (24 scores of 30.5 and 10 of 31.5); these values were present in the measurement data gathered by the clinic and represent data quality anomalies of undetermined origin. Clipping MNA values to 0–30 would prevent impossible scores but introduce boundary effects that we have avoided by keeping these data in the dataset. All 388 stored categories agree with the available total-score thresholds. Independent refitting of the primary logistic and core continuous-score models using the de-identified numerical data reproduced the reported estimates and confidence intervals. Sensitivity analyses excluding these observations or capping them at 30 are reported in Supplementary Table S3. Finally, two variables corresponding to MNA items, namely living arrangements (item G) and polypharmacy (item H), showed labelling or coding inconsistencies and are components of the outcome; they were therefore not used as predictors. A separately supplied corrected MNA total variable was not adopted because it contained a scaling anomaly, and the categorical MNA classification, which was stable across linked datasets, was used throughout.

### 2.6. Statistical Analysis

Analyses were organised into a hierarchy. The primary analysis was a multivariable logistic regression for the binary outcome (at risk versus normal). To limit criterion overlap with the MNA, predictors were restricted to variables assessed separately from the MNA and were chosen a priori on clinical and literature grounds, though not pre-registered: age, sex, depressive symptoms, handgrip strength and education. This covariate set was defined before fitting models for the present nutritional outcome. Handgrip strength was selected as the primary physical-function measure because it is an objective and clinically established measure of muscle strength, had high data completeness, and did not depend on the gait-distance derivation that required correction; gait speed and SPPB were evaluated as sensitivity substitutions. Because three-level education contributes two parameters, the model contained six parameters (approximately 7.7 events per parameter); we therefore also fitted a Firth penalised logistic regression as a small-sample sensitivity analysis. Body mass index was not entered because it is embedded within the MNA. Secondary analyses used the continuous MNA score in linear regression with the same predictors, with HC3 robust standard errors given a bounded, ceiling-affected outcome, and added, in turn, serum 25-hydroxyvitamin D and body composition (VAT and whole-body fat mass); to separate covariate adjustment from population change, the core model was refitted on each sensitivity subset. Exploratory analyses added HDL cholesterol and triglycerides, examined the prevalence of nutritional risk across tertiles of VAT, body mass index and total fat mass (with 95% confidence intervals and a trend test), tested VAT in the binary model with and without adjustment for depressive symptoms and stratified by sex, and visualised the additive joint pattern of the two dominant continuous correlates as a response surface (a GDS-by-VAT interaction was formally tested). Descriptive comparisons used the Mann–Whitney U test for continuous variables and the chi-squared or Fisher exact test for categorical variables. Model diagnostics comprised logit linearity, multicollinearity (variance inflation factors), influence (Cook’s distance) and homoscedasticity. Analyses were complete-case (variable-level availability is reported in Supplementary Table S2); estimates are reported with 95% confidence intervals; two-sided *p*-values below 0.05 were considered significant but, given the exploratory design, were interpreted descriptively and not adjusted for multiplicity. Analyses were performed in Python 3.12.13 (statsmodels 0.14.6, scipy 1.17.1, matplotlib 3.10.9) To examine selection related to incomplete MNA availability, stabilised inverse-probability weights were estimated from age, sex, body mass index and GDS-15; weighted prevalence and the weighted primary model were compared with their unweighted counterparts. Natural cubic splines with a three-column basis (Patsy cr(x, *df* = 3), without a centring constraint) tested departures from linearity for age, GDS-15, handgrip strength and VAT. The basis spans a constant component already represented by the model intercept, leaving two independent predictor components; compared with a single linear term, the spline therefore adds one parameter, and each likelihood-ratio test of non-linearity has one degree of freedom. Handgrip was additionally standardised within sex, and the GDS association was expressed for a three-point contrast and as model-based absolute risks at representative scores.

## 3. Results

### 3.1. Participants and Prevalence of Nutritional Risk

The analytic sample comprised 388 community-dwelling older adults with a complete MNA. The mean age was 71.5 years (standard deviation 5.6), and 286 (73.7%) were women. The mean body mass index was 29.7 kg/m^2^ (standard deviation 4.9), indicating an on average overweight cohort. No participant met the criterion for established malnutrition. Fifty-two participants (13.4%, 95% CI 10.2 to 17.2) were classified as being at risk of malnutrition and 336 (86.6%) as normal. The comorbidity burden was low, with a median Charlson index of 0. Participants were self-selected volunteers; compared with the 311 cohort members without a complete MNA (Supplementary Table S1) they were largely similar, including on all principal predictors (GDS *p* = 0.76, education *p* = 0.54, VAT *p* = 0.85, body mass index *p* = 0.97), but were marginally older (median 71 vs. 69 years, *p* = 0.008) and had slightly lower MoCA (28 vs. 29, *p* = 0.001) and SPPB (9 vs. 10, *p* = 0.022). In a selection model, MNA availability was associated with age (odds ratio 1.04 per year, 95% CI 1.01 to 1.07; *p* = 0.007), but not with sex, body mass index or GDS-15. Inverse-probability weighting yielded an at-risk prevalence of 13.2% and a GDS-15 odds ratio of 1.29 (95% CI 1.18 to 1.42), closely matching the unweighted results (Supplementary Table S4). Excluding the one participant recorded as age 59 who had a complete MNA left 387 participants and 51 at-risk events; the primary model included 341 participants and 45 events, and the GDS-15 estimate was unchanged (odds ratio 1.29, 95% CI 1.18 to 1.42; Supplementary Table S12).

Table 1 compares participants by MNA category. Those at risk of malnutrition had substantially higher depressive symptom scores (median GDS 6 vs. 2, *p* < 0.001) and SARC-F scores (3.5 vs. 2, *p* < 0.001), slower gait speed (0.68 vs. 0.81 m/s, *p* < 0.001), a lower SPPB (8 vs. 9, *p* < 0.001), lower HDL cholesterol (1.17 vs. 1.46 mmol/L, *p* < 0.001) and higher triglycerides (1.76 vs. 1.34 mmol/L, *p* = 0.03). Age, sex, body mass index, handgrip strength, appendicular lean mass index, VAT, total fat mass, MoCA, vitamin D, total and LDL cholesterol, glucose, comorbidity and marital status did not differ significantly between groups. Lower education was more common among those at risk (37% vs. 22% of the normal group), although this did not reach significance (*p* = 0.08).

### 3.2. Primary Model: Correlates of Nutritional Risk

**Figure 1:**
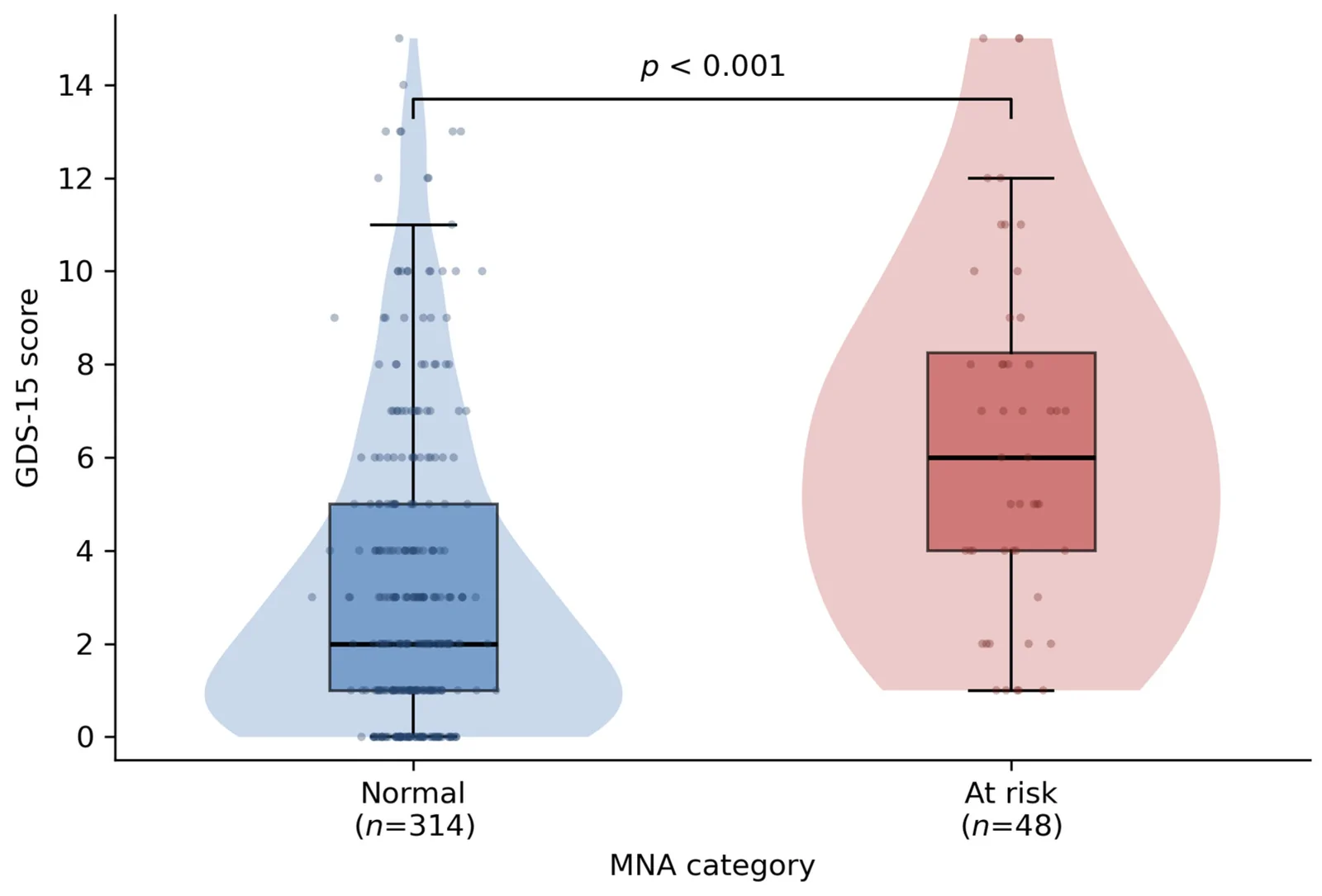
Distribution of GDS-15 scores by MNA category among 362 participants with both measurements available (normal, *n* = 314; at risk of malnutrition, *n* = 48). Violin plots show distributions, boxes show medians and interquartile ranges, and points show individual observations; the *p*-value is from the Mann–Whitney U test. GDS-15, 15-item Geriatric Depression Scale; MNA, Mini Nutritional Assessment.

In the multivariable logistic regression (342 participants with complete data, 46 at-risk events), depressive symptoms were the dominant correlate of nutritional risk (Table 2). Each one-point increase in the GDS-15 was associated with 29% higher odds of nutritional risk (odds ratio 1.29, 95% CI 1.18 to 1.41), corresponding to an odds ratio of 2.14 (95% CI 1.63 to 2.82) for a 3-point increase. Lower education was associated with approximately twice the odds of nutritional risk relative to secondary education, although the confidence interval crossed the null (odds ratio 2.08, 95% CI 0.95 to 4.56); this association was therefore suggestive rather than conclusive for the binary outcome. Age, sex and handgrip strength were not independently associated with nutritional risk. The model was statistically significant overall (likelihood-ratio *p* < 0.001; McFadden pseudo R-squared 0.15). A Firth penalised logistic regression, fitted because the model contained six parameters for 46 events, produced essentially identical estimates (GDS odds ratio 1.28, 95% CI 1.17 to 1.40), giving little evidence that the primary estimate was materially affected by small-sample bias. The marked separation in depressive symptom scores between the two MNA categories is shown in Figure 1.

Model-based risk increased from 4.2% (95% CI 2.0 to 8.4) at GDS 1 to 13.4% (8.1 to 21.4) at GDS 6 and 25.0% (15.3 to 38.1) at GDS 9 (Supplementary Table S7). Within-sex standardised handgrip was not independently associated with risk (odds ratio 1.38 per standard deviation, 95% CI 0.96 to 1.98; *p* = 0.084) (Supplementary Table S6). Tests using restricted cubic splines did not show significant non-linearity for age (*p* = 0.96), GDS-15 (*p* = 0.07) or handgrip strength (*p* = 0.23) (Supplementary Table S5 and Supplementary Figure S2).

### 3.3. Secondary and Sensitivity Analyses

The linear regression on the continuous MNA score, which retains the information in the full score, was consistent with the primary model (Table 3) and is reported with HC3 robust standard errors given a ceiling-affected outcome. Higher GDS was associated with a lower MNA score (coefficient −0.32 per point, 95% CI −0.40 to −0.24), and lower education was now significantly associated with a lower MNA score (coefficient −0.82, 95% CI −1.46 to −0.19). The model explained about one-fifth of the variance in the MNA score (adjusted R-squared 0.20). Adding serum 25-hydroxyvitamin D (259 participants) did not materially change the depression or education associations, and vitamin D itself was only weakly and non-significantly associated with the MNA score (coefficient 0.008 per nmol/L, 95% CI −0.006 to 0.023).

When body composition was added (309 participants), depressive symptoms remained strongly associated with the MNA score, and higher VAT was independently associated with a lower MNA score (coefficient −0.51, 95% CI −0.99 to −0.04), whereas whole-body fat mass was not. In this model, higher VAT, but not whole-body fat mass, was associated with a lower MNA score. Refitting the core model on each sensitivity subset gave stable coefficients (GDS b −0.33 to −0.29; lower-education b −1.15 to −0.77), indicating that these associations were not artefacts of the changing complete-case population (Supplementary Table S8).

In a further sensitivity model that added the lipid fractions that had differed at the bivariate level (179 participants), higher HDL cholesterol was independently associated with a higher MNA score (coefficient 1.25 per mmol/L, 95% CI 0.25 to 2.24), whereas triglycerides were not independently associated once HDL and the other covariates were included. Depressive symptoms and lower education retained their associations in this smaller subsample. A parallel logistic model was directionally consistent, with higher HDL associated with lower odds of nutritional risk, with an odds ratio of 0.04 (95% CI 0.005 to 0.33; *p* = 0.003), whereas triglycerides were not associated (odds ratio 0.95, 95% CI 0.48 to 1.90; *p* = 0.89); these estimates remain exploratory because the model included only 22 events. Because the lipid subsample was less than half the core sample, these findings are regarded as exploratory (Supplementary Table S10).

A formal GDS-by-VAT interaction was non-significant (*p* = 0.72). For completeness, the additive joint pattern of the two dominant continuous correlates is provided as an exploratory response surface in Supplementary Figure S1, on which the predicted MNA score is highest when both depressive symptoms and visceral adiposity are low.

### 3.4. Nutritional Risk and Adiposity

Nutritional risk was not concentrated in the leanest participants. In a logistic model for the binary outcome adjusted for age and sex, higher VAT was associated with at-risk status (odds ratio 1.46 per kg, 95% CI 1.02 to 2.10, *p* = 0.038); the bivariate non-significance reflected confounding by sex, since men had substantially higher VAT (median 2.00 vs. 1.26 kg). This association attenuated to non-significance after adjustment for depressive symptoms (odds ratio 1.34, 95% CI 0.91 to 1.99, *p* = 0.14). In exploratory sex-stratified analyses the association was present in men (VAT 2.55 vs. 1.91 kg, *p* = 0.038) but not women (1.25 vs. 1.34 kg, *p* = 0.56); however, a formal VAT-by-sex interaction was non-significant (*p* = 0.27), only 14 of the 102 men were at risk, and the stratified analysis is therefore underpowered, so the difference between sexes should not be over-interpreted. The prevalence of nutritional risk rose across tertiles of VAT (10.8% [95% CI 6.5 to 17.3], 11.6% [7.2 to 18.3] and 17.8% [12.2 to 25.3]; trend *p* = 0.098) (Supplementary Table S9) and of body mass index (12.3%, 11.5% and 16.4%). Taken together, these results indicate that nutritional risk coincided with, rather than excluded, higher visceral adiposity, but as a non-independent, exploratory and hypothesis-generating signal that requires independent replication. Comorbidity (Charlson index) and marital status were not associated with nutritional risk, and adding them to the primary model did not improve it.

### 3.5. Sensitivity Analyses and Model Diagnostics

Findings were robust across sensitivity analyses. Replacing handgrip with gait speed (odds ratio 0.18 per m/s, 95% CI 0.03 to 1.07, *p* = 0.060) or with SPPB (odds ratio 0.82 per point, 95% CI 0.67 to 1.02, *p* = 0.070) left the depression association unchanged (GDS odds ratio 1.23 to 1.29, *p* < 0.001) and no performance measure was clearly independent. Diagnostics supported the models: variance inflation factors were 1.06 to 1.09 (no collinearity); a squared GDS term was non-significant (*p* = 0.069), with no strong evidence against approximate linearity; no observation was influential (maximum Cook’s distance 0.03). The continuous MNA score showed a ceiling (24% of scores at or above 29) and heteroscedasticity (Breusch-Pagan *p* = 0.008), so linear estimates are reported with HC3 robust standard errors, under which the depression and education associations were retained (GDS b −0.32, 95% CI −0.40 to −0.24; lower-education b −0.82, 95% CI −1.46 to −0.19).

## 4. Discussion

In this community sample of older adults attending sarcopenia screening, no participant met the criterion for established malnutrition and approximately one in seven was at risk of malnutrition. The single most prominent correlate of MNA-defined nutritional risk was the burden of depressive symptoms, which was consistent and of substantial magnitude across both the logistic model for nutritional risk and the secondary continuous-score model. Lower education showed a weaker, less consistent association, suggestive for the binary outcome and significant only for the continuous score. Neither leanness nor low body mass characterised those at risk; an association with higher visceral adiposity was exploratory and, on stratified analysis, was seen in men but not women without formal evidence of effect modification.

The prominence of depressive symptoms is consistent with a substantial literature linking low mood with reduced appetite, diminished interest in food preparation and social eating, and weight change in later life [8,9]. Because the analysis is cross-sectional, the direction of this association cannot be established; poor nutrition and depression plausibly reinforce one another. The practical implication, appropriate to a cross-sectional design, is that nutritional screening in this population may be incomplete without concurrent evaluation of mood.

This central finding must, however, be read alongside a conceptual limitation that we consider its most important caveat. The MNA itself contains a neuropsychological item that captures dementia and depression, so the depression measure and the nutritional outcome are not fully independent constructs, even though the GDS-15 was administered separately. Part of the observed association is therefore likely to reflect genuine clinical coexistence of low mood and poor nutrition, and part may reflect criterion overlap between the two instruments; the present dataset, which lacks item-level MNA responses, cannot quantify the relative contribution of these two mechanisms. This overlap may extend beyond the explicit neuropsychological item, since depressive symptoms may also track the MNA items concerning appetite, food intake and self-perceived nutritional status, and even a small shared component can affect classification for individuals whose scores lie close to the MNA threshold. Readers should therefore not interpret the odds ratio of 1.29 as a wholly external correlate of nutritional status. For this reason, we describe depressive symptoms throughout as a correlate rather than a determinant of nutritional risk.

The educational gradient echoes the wider social patterning of nutritional risk, whereby lower educational attainment is associated with poorer nutritional status through pathways that may include health literacy, material resources and dietary habits [10]. The association was significant only for the continuous score and did not follow a monotonic ordinal trend (coefficient 0.20 per education level; *p* = 0.21; Supplementary Table S10), suggesting that any association may be concentrated in the lowest education category; it should be regarded as suggestive.

A clinically salient observation is that nutritional risk was not confined to participants with low body mass. After adjustment for age and sex, higher VAT was associated with at-risk status, although this attenuated after adjusting for depressive symptoms and, in underpowered stratified analyses with only 14 male events, was seen in men but not women without formal evidence of effect modification; it is best regarded as an exploratory, hypothesis-generating signal rather than an independent correlate. Flexible modelling did not identify a non-linear VAT association (*p* = 0.73). This pattern cautions against the assumption that a normal or high body mass excludes nutritional concern, and aligns with the broader recognition that adiposity and nutritional or muscular deficits can coexist [12]. This is relevant in a region where overweight and obesity are frequently reported in older adults [21,22], and where body mass index alone may conceal risk-carrying patterns of adipose-tissue distribution such as normal-weight or abdominal obesity [12]. It also underlines the conceptual distinction, increasingly formalised in the GLIM framework, between risk of malnutrition as screened by the MNA and diagnosed malnutrition [23]; the present findings pertain to MNA-defined risk, not to diagnosed malnutrition, of which there were no cases. It suggests that older adults who appear adequately or over-nourished should not be excluded from nutritional screening.

Several variables commonly implicated in nutritional risk were not associated with it here. Handgrip strength, appendicular lean mass, vitamin D, the comorbidity burden and marital status showed no significant independent association [7,11]. The low comorbidity burden, the near-absence of established malnutrition and the relatively preserved physical performance together portray a comparatively healthy, self-selected community sample, in which the range of these exposures may have been too narrow to reveal associations that are apparent in frailer populations. Recent community-based studies continue to document substantial malnutrition risk and its social and health determinants in older adults [24]. Among the lipid fractions, higher HDL cholesterol was associated with better nutritional status in a subsample of fewer than 180 participants; this exploratory finding warrants cautious interpretation.

### Strengths and Limitations

Strengths include the use of a widely validated nutritional assessment instrument, albeit without a formally validated Serbian-language version (see below), objectively measured body composition including visceral fat by dual-energy X-ray absorptiometry, and a deliberate analytic strategy that distinguished correlates assessed separately from the MNA from items embedded within it, while explicitly addressing the remaining conceptual overlap between depressive symptoms and the MNA. Data quality issues were identified and addressed transparently, including correction of the gait and SPPB measurements and exclusion of implausible values.

The limitations are important. The design is cross-sectional, so no causal or temporal inference is warranted, and the language throughout is deliberately associational. The full MNA was available for about 55% of the source cohort, and the analysis is complete-case; if the availability of an MNA was related to nutritional status, selection could bias the prevalence estimate and, less predictably, the associations. Inverse-probability-weighted estimates were similar, but this analysis assumes that selection was adequately represented by the observed age, sex, body mass index and GDS-15 variables. No participant met the criterion for established malnutrition, so the findings pertain to the earlier at-risk phase and may not generalise to frailer or institutional populations. Serum albumin, prealbumin and glycated haemoglobin were not available [1], and C-reactive protein and serum iron were too sparse to use, so an inflammatory or glycaemic dimension could not be examined. Comorbidity was captured by the Charlson index, which was low across the cohort and may under-represent the burden relevant to nutrition. The lipid, glucose and vitamin D subsamples were smaller than the core sample, so the corresponding analyses are secondary. Cohort linkage relied on non-identifying keys and introduced the scaling artefacts described in the Methods, which were addressed but which underline the value of the cleaning steps taken. In addition, the cohort was a self-selected volunteer sample recruited through pensioners’ associations, which limits generalisability and probably explains the absence of established malnutrition and the low comorbidity burden; the analysis was exploratory and not pre-registered; item-level MNA data were unavailable, so the neuropsychological item could not be removed to test criterion overlap directly; and although the GDS-15 was used in a validated Serbian version, no validated Serbian version of the MNA outcome itself, or of the other instruments, has been published. The continuous-score analyses were affected by a ceiling, heteroscedasticity and above-maximum values of undocumented origin; capping and exclusion analyses were stable, but these models remain secondary.

## 5. Conclusions

In this relatively preserved volunteer cohort, depressive symptoms were the most robust correlate of MNA-defined malnutrition risk, although the interpretation is tempered by conceptual overlap because the MNA includes a depression/dementia item. Lower education showed a weaker association, while the observed relationship with visceral adiposity was exploratory and appeared, in underpowered stratified analyses, in men but not women. Nutritional risk was not confined to participants with low body mass. These cross-sectional, associational findings support nutritional screening that is coupled with evaluation of mood, and that does not exclude older adults with normal or high body mass, but they require confirmation in independent cohorts.

## Figures and Tables

**Table 1:** Characteristics of the analytic sample by MNA category (*n* = 388).

Characteristic	Normal (*n* = 336)	At Risk (*n* = 52)	*p*
Age, years	70.5 (67.0 to 74.3)	72.0 (68.8 to 77.0)	0.12
Women, *n* (%)	248 (74%)	38 (73%)	1.00
Body mass index, kg/m^2^	29.2 (26.3 to 32.2)	29.9 (26.3 to 33.1)	0.49
Gait speed, m/s	0.81 (0.67 to 1.00)	0.68 (0.51 to 0.85)	<0.001
SPPB total (0 to 12)	9 (8 to 11)	8 (6 to 9)	<0.001
Handgrip strength, kg	26 (20 to 32)	26 (20 to 34)	0.82
ALM/height2, kg/m^2^	7.29 (6.61 to 8.04)	7.34 (6.65 to 8.69)	0.43
VAT mass, kg	1.42 (0.90 to 1.98)	1.57 (1.05 to 2.41)	0.12
Total fat mass, kg	30.4 (24.5 to 36.9)	30.7 (24.2 to 37.8)	0.86
SARC-F (0 to 10)	2 (1 to 4)	3.5 (2 to 6)	<0.001
GDS-15 (0 to 15)	2 (1 to 5)	6 (4 to 8)	<0.001
MoCA (0 to 30)	28 (26 to 29)	28 (26 to 29)	0.83
25-hydroxyvitamin D, nmol/L	50 (35 to 65)	51.5 (42 to 67.5)	0.58
Total cholesterol, mmol/L	5.69 (4.79 to 6.28)	5.24 (4.61 to 5.92)	0.18
HDL cholesterol, mmol/L	1.46 (1.29 to 1.71)	1.17 (1.07 to 1.37)	<0.001
Triglycerides, mmol/L	1.34 (1.00 to 1.86)	1.76 (1.31 to 1.94)	0.03
Glucose, mmol/L	5.80 (5.25 to 6.60)	5.70 (5.10 to 6.67)	0.64
Charlson index	0 (0 to 1)	0 (0 to 1)	0.54
Lower education, *n* (%)	75 (22%)	19 (37%)	0.08

Values are median (interquartile range) unless stated. Continuous variables were compared with the Mann–Whitney U test; categorical variables with the chi-squared or Fisher exact test. ALM, appendicular lean mass; GDS, Geriatric Depression Scale; MoCA, Montreal Cognitive Assessment; SPPB, Short Physical Performance Battery; VAT, visceral adipose tissue.

**Table 2:** Multivariable logistic regression for risk of malnutrition (at-risk versus normal); *n* = 342, 46 events.

Predictor	Odds Ratio	95% CI	*p*
Age (per year)	1.03	0.97 to 1.10	0.29
Male sex	0.82	0.27 to 2.43	0.72
GDS-15 (per point)	1.29	1.18 to 1.41	<0.001
Handgrip strength (per kg)	1.04	0.99 to 1.10	0.11
Education: lower vs. secondary	2.08	0.95 to 4.56	0.07
Education: higher vs. secondary	1.43	0.56 to 3.66	0.46

Reference categories are female sex and completed secondary school. Predictors were assessed separately from the MNA instrument; conceptual overlap between GDS and the MNA is addressed in the Discussion. GDS, Geriatric Depression Scale.

**Table 3:** Linear regression on the continuous MNA score: secondary and sensitivity models (regression coefficients).

Predictor	Core (*n* = 342)	+Vitamin D (*n* = 259)	+Body Composition (*n* = 309)	+Lipids (*n* = 179)
GDS-15 (per point)	−0.32 (−0.40, −0.24) *	−0.34 (−0.43, −0.24) *	−0.29 (−0.37, −0.20) *	−0.28 (−0.39, −0.17) *
Education: lower vs. secondary	−0.82 (−1.46, −0.19) *	−0.94 (−1.67, −0.21) *	−0.76 (−1.44, −0.07) *	−1.11 (−1.96, −0.25) *
Handgrip (per kg)	0.01 (−0.03, 0.04)	0.00 (−0.03, 0.04)	0.02 (−0.01, 0.06)	−0.01 (−0.06, 0.03)
25-OH vitamin D (per nmol/L)	-	0.008 (−0.006, 0.023)	-	-
VAT mass (per kg)	-	-	−0.51 (−0.99, −0.04) *	-
Total fat mass (per kg)	-	-	0.01 (−0.03, 0.05)	-
HDL cholesterol (per mmol/L)	-	-	-	1.25 (0.25, 2.24) *
Triglycerides (per mmol/L)	-	-	-	0.11 (−0.28, 0.49)
Adjusted R-squared	0.20	0.22	0.19	0.23

Values are regression coefficients (95% confidence intervals) with HC3 robust standard errors. All models adjusted for age and sex. An asterisk denotes *p* < 0.05. VAT, visceral adipose tissue; GDS, Geriatric Depression Scale.

## Data Availability

De-identified data may be available from the corresponding author on reasonable request, subject to institutional approval.

## Supplementary Materials

The following supporting information can be downloaded at: https://www.mdpi.com/article/10.3390/nu18213478/s1.
