# Supplementary material for: Malnutrition Risk and Depressive Symptoms in Community-Dwelling Older Adults
Source: Nutrients. 2026 Oct 23;18(21):3478. doi: 10.3390/nu18213478 (PMC13764706; doi:10.3390/nu18213478)
Supplement: Supplementary file 1 [file nutrients-18-03478-s001.zip › nutrients-4601902-supplementary.pdf]

Supplementary material

Malnutrition risk and depressive symptoms in community-dwelling older adults - revised

Supplementary Table S1. *Comparison of participants with versus without a complete MNA (selection analysis).*

Values are median (interquartile range) unless stated. Continuous variables compared with the Mann-Whitney U test; categorical variables with the chi-squared or Fisher exact test. ALM, appendicular lean mass; GDS, Geriatric Depression Scale; MoCA, Montreal Cognitive Assessment; SPPB, Short Physical Performance Battery; VAT, visceral adipose tissue.

Characteristic	With MNA (n = 388)	Without MNA (n = 311)	p
Age, years	71.0 (67 to 75)	69.0 (66 to 73)	0.008
Women, n (%)	286 (74%)	222 (71%)	0.55
Body mass index, kg/m2	29.3 (26.3 to 32.2)	29.6 (26.1 to 32.7)	0.97
GDS-15 (0 to 15)	3 (1 to 6)	2 (1 to 5)	0.76
MoCA (0 to 30)	28 (26 to 29)	29 (27 to 30)	0.001
Gait speed, m/s	0.80 (0.6 to 1.0)	0.90 (0.7 to 1.0)	0.09
SPPB total (0 to 12)	9 (8 to 11)	10 (8 to 11)	0.022
Handgrip strength, kg	26 (20 to 32)	26 (21 to 34)	0.37
ALM/height2, kg/m2	7.3 (6.6 to 8.1)	7.5 (6.6 to 8.3)	0.15
VAT mass, kg	1.4 (0.9 to 2.0)	1.3 (0.9 to 2.1)	0.85
Total fat mass, kg	30.5 (24.5 to 37.0)	30.4 (24.5 to 35.5)	0.60
25-hydroxyvitamin D, nmol/L	51 (36 to 65)	48 (35 to 64)	0.40
Charlson index	0 (0 to 1)	0 (0 to 1)	0.92
Lower education, n (%)	94 (24%)	77 (25%)	0.54

Values are median (IQR); denominators are variable-specific.

Supplementary Table S2. *Availability of each variable within the analytic sample (n = 388).*

Analyses were complete-case. The lipid, glucose and vitamin D variables were the least complete, so the corresponding models are secondary or exploratory. ALM, appendicular lean mass; GDS, Geriatric Depression Scale; MoCA, Montreal Cognitive Assessment; SPPB, Short Physical Performance Battery; VAT, visceral adipose tissue.

Variable	Available / 388	Missing, %
Age; sex; body mass index	388	0.0
Education	374	3.6
GDS-15	362	6.7
MoCA	273	29.6
Handgrip strength (cleaned)	378	2.6
Gait speed (8 m)	373	3.9
SPPB (corrected)	351	9.5
VAT mass; ALM/height2	388	0.0
Total fat mass (cleaned)	350	9.8
25-hydroxyvitamin D	297	23.5
Total cholesterol	208	46.4

Variable	Available / 388	Missing, %
HDL cholesterol	206	46.9
Triglycerides	207	46.6
Glucose	193	50.3
Charlson index	341	12.1
Marital status	349	10.1

Supplementary Table S3. *Sensitivity of the secondary continuous-MNA associations to the 34 scores above the instrument maximum.*

Linear regression on the continuous MNA score, adjusted for age, sex and handgrip strength. Excluding or capping the 34 values marginally above the maximum did not materially change the depression or education associations. GDS, Geriatric Depression Scale.

Analysis	n	GDS-15 coefficient (p)	Lower-education coefficient (p)
All observations	342	-0.32 (p < 0.001)	-0.82 (p = 0.009)
Excluding MNA > 30	312	-0.25 (p < 0.001)	-0.74 (p = 0.016)
Capping MNA at 30	342	-0.30 (p < 0.001)	-0.78 (p = 0.010)

Supplementary Table S4. *Inverse-probability-weighted primary model*

Stabilised inverse-probability weights were estimated from a logistic model for availability of a complete MNA using age, sex, body mass index and GDS-15 among 647 participants with complete selection-model covariates. The weighted primary logistic regression used the same outcome and predictors as the unweighted primary model. Values are odds ratios with 95% confidence intervals. Female sex and completed secondary education are the reference categories. The weighted prevalence of MNA-defined nutritional risk was 13.2%; the effective sample size based on the observed weights was 358.5. GDS, Geriatric Depression Scale; MNA, Mini Nutritional Assessment.

Predictor	Odds ratio	95% CI	p
Male sex	0.78	0.28 to 2.19	0.637
Education: lower vs secondary	2.06	0.93 to 4.57	0.075
Education: higher vs secondary	1.49	0.58 to 3.85	0.407
Age (per year)	1.03	0.96 to 1.11	0.396
GDS-15 (per point)	1.29	1.18 to 1.42	<0.001
Handgrip strength (per kg)	1.04	0.99 to 1.10	0.081

Supplementary Table S5. *Restricted cubic-spline tests*

Natural cubic splines with three degrees of freedom were compared with the corresponding linear terms using likelihood-ratio tests. Age, GDS-15 and handgrip strength were evaluated in the primary logistic model; VAT was evaluated in a model adjusted for age, sex and GDS-15. The p values test departure from a linear association and provide no strong evidence of non-linearity for any evaluated predictor. GDS, Geriatric Depression Scale; VAT, visceral adipose tissue.

Predictor	Model	n	Likelihood-ratio chi-squared	df	p for non-linearity
Age	Primary	342	0.00	1	0.960
GDS-15	Primary	342	3.28	1	0.070
Handgrip strength	Primary	342	1.46	1	0.227
VAT mass	Age-, sex- and GDS-adjusted VAT model	362	0.12	1	0.730

Supplementary Table S6. *Within-sex standardised handgrip*

Handgrip strength was standardised separately within women and men before fitting the primary logistic model, thereby expressing the estimate per sex-specific standard deviation while retaining adjustment for age, sex, GDS-15 and education. Values are odds ratios with 95% confidence intervals. Female sex and completed secondary education are the reference categories. GDS, Geriatric Depression Scale.

Contrast	Odds ratio	95% CI	p	n
Per within-sex standard deviation increase in handgrip strength	1.38	0.96 to 1.98	0.084	342

Supplementary Table S7. *Clinically scaled GDS association*

The primary logistic model was used to express the GDS-15 association over a clinically interpretable three-point contrast and as absolute predicted risks. Predicted risks refer to a 71-year-old woman with completed secondary education and handgrip strength of 26 kg, with all other model terms held as specified. Values are odds ratios or predicted probabilities with 95% confidence intervals. GDS, Geriatric Depression Scale.

Contrast	Estimate	95% CI
3-point higher GDS-15	OR 2.14	1.63 to 2.82
Predicted risk at GDS=1	4.2%	2.0% to 8.4%
Predicted risk at GDS=3	6.7%	3.7% to 12.1%
Predicted risk at GDS=6	13.4%	8.1% to 21.4%
Predicted risk at GDS=9	25.0%	15.3% to 38.1%

Supplementary Table S8. *Core-model refits within each complete-case sensitivity subset.*

The same core linear model was refitted without the added covariates on each complete-case subset. Values are coefficients with HC3 robust 95% confidence intervals. GDS, Geriatric Depression Scale.

Complete-case subset	n	GDS-15 coefficient (95% CI)	Lower-education coefficient (95% CI)
Core	342	-0.32 (-0.40 to -0.24)	-0.82 (-1.46 to -0.19)
Vitamin D subset	259	-0.33 (-0.43 to -0.24)	-0.97 (-1.69 to -0.25)
Body-composition subset	309	-0.30 (-0.38 to -0.21)	-0.77 (-1.45 to -0.09)
Lipid subset	179	-0.29 (-0.40 to -0.18)	-1.15 (-2.01 to -0.30)

Supplementary Table S9. *Exploratory adiposity, stratified, and interaction analyses.*

Tertile rows report the prevalence of MNA-defined nutritional risk with Wilson 95% confidence intervals and a linear trend test. Sex-stratified rows compare median VAT mass using the Mann-Whitney U test. The VAT-by-sex row is from the age-adjusted binary model; the GDS-by-VAT row is from the age- and sex-adjusted continuous-MNA model with HC3 robust standard errors. BMI, body mass index; GDS, Geriatric Depression Scale; MNA, Mini Nutritional Assessment; OR, odds ratio; VAT, visceral adipose tissue.

Analysis	n	Estimate	95% CI / comparison	p
VAT tertile 1	130	10.8%	6.5% to 17.3%	
VAT tertile 2	129	11.6%	7.2% to 18.3%	
VAT tertile 3	129	17.8%	12.2% to 25.3%	0.098 (trend)
BMI tertile 1	130	12.3%	7.7% to 19.1%	
BMI tertile 2	130	11.5%	7.1% to 18.2%	
BMI tertile 3	128	16.4%	11.0% to 23.8%	0.337 (trend)
Fat-mass tertile 1	117	16.2%	10.6% to 24.0%	

Analysis	n	Estimate	95% CI / comparison	p
Fat-mass tertile 2	116	9.5%	5.4% to 16.2%	
Fat-mass tertile 3	117	15.4%	10.0% to 23.0%	0.849 (trend)
VAT: women	286	1.25 vs 1.34 kg	Normal vs at risk	0.559
VAT: men	102	1.91 vs 2.55 kg	Normal vs at risk	0.038
VAT-by-sex interaction	388	OR 1.51	0.73 to 3.14	0.271
GDS-by-VAT interaction	330	b = 0.020	-0.086 to 0.125	0.715

Supplementary Table S10. *Education trend and additional logistic models.*

The education trend is from the continuous-MNA model adjusted for age, sex and GDS-15. Other rows add the stated covariate or covariate set to the primary logistic model; likelihood-ratio p values compare the expanded and core models on the same complete-case subset. The lipid model contained 22 at-risk events and is exploratory. CI, confidence interval; HDL, high-density lipoprotein; LR, likelihood ratio; MNA, Mini Nutritional Assessment; OR, odds ratio.

Analysis	n	Estimate	95% CI	Term p	Model LR p
Education ordinal trend	350	b = 0.20	-0.11 to 0.51	0.210	-
Charlson index	301	OR 1.08	0.61 to 1.92	0.789	0.791
Marital status	306	-	-	-	0.313
Charlson + marital status	278	-	-	-	0.539
HDL cholesterol	179	OR 0.04	0.005 to 0.33	0.003	0.001
Triglycerides	179	OR 0.95	0.48 to 1.90	0.888	0.001

Supplementary Table S11. *Data-quality review, cleaning actions and analytic consequences.*

Counts distinguish the 699-person source cohort from the 388-person complete-MNA analytic sample. Available counts are shown for the recalculated gait and SPPB variables because the distance-processing correction applied to all observations with sufficient source measurements. Values without a defensible correction were set to missing rather than imputed. BMI, body mass index; MNA, Mini Nutritional Assessment; SPPB, Short Physical Performance Battery.

Issue	Source cohort	Analytic sample	Action and sensitivity check
Gait course processed as 4 m instead of the administered 8 m	659 recalculable	373 recalculable	Recalculated speed over 8 m; corrected median 0.81 m/s; gait substitution model reported
SPPB gait component affected by distance processing	619 rescorable	351 rescorable	Rescored SPPB using corrected gait component; corrected median 9; SPPB substitution model reported
Physically implausible handgrip values (>90 kg)	2	1	Set to missing; within-sex standardised and alternative performance models reported
Whole-body fat mass <5 kg and inconsistent with BMI	69	38	Set to missing for fat-mass analyses; VAT and BMI analyses retained separately
Continuous MNA score above the 30-point ceiling (30.5 or 31.5)	34	34	Category unchanged; exclusion and capping sensitivities reported in Table S3
Living-arrangement and polypharmacy variables corresponding to MNA items	Variable-level coding inconsistency	Variable-level coding inconsistency	Not used as predictors because coding was inconsistent and the items form part of the outcome
Alternative corrected MNA total	Scaling anomaly	Scaling anomaly	Not used; stable categorical MNA classification retained

Supplementary Table S12. *Primary-model sensitivity excluding the participant recorded as age 59.*

Two members of the 699-person source cohort were recorded as age 59 but were within two months of their 60th birthdays; only one had a complete MNA and entered the analytic sample. After excluding that participant, the analytic sample contained 387 participants and 51 at-risk events; the complete-case primary model contained 341 participants and 45 events. Values are odds ratios with 95% confidence intervals. Female sex and completed secondary education are the reference categories. GDS, Geriatric Depression Scale; MNA, Mini Nutritional Assessment.

Predictor	Odds ratio	95% CI	p
Age (per year)	1.05	0.98 to 1.12	0.158
Male sex	0.82	0.27 to 2.47	0.723
GDS-15 (per point)	1.29	1.18 to 1.42	<0.001
Handgrip strength (per kg)	1.05	0.99 to 1.10	0.084
Education: lower vs secondary	2.23	1.00 to 4.93	0.049
Education: higher vs secondary	1.51	0.58 to 3.92	0.395

Supplementary Figure S1

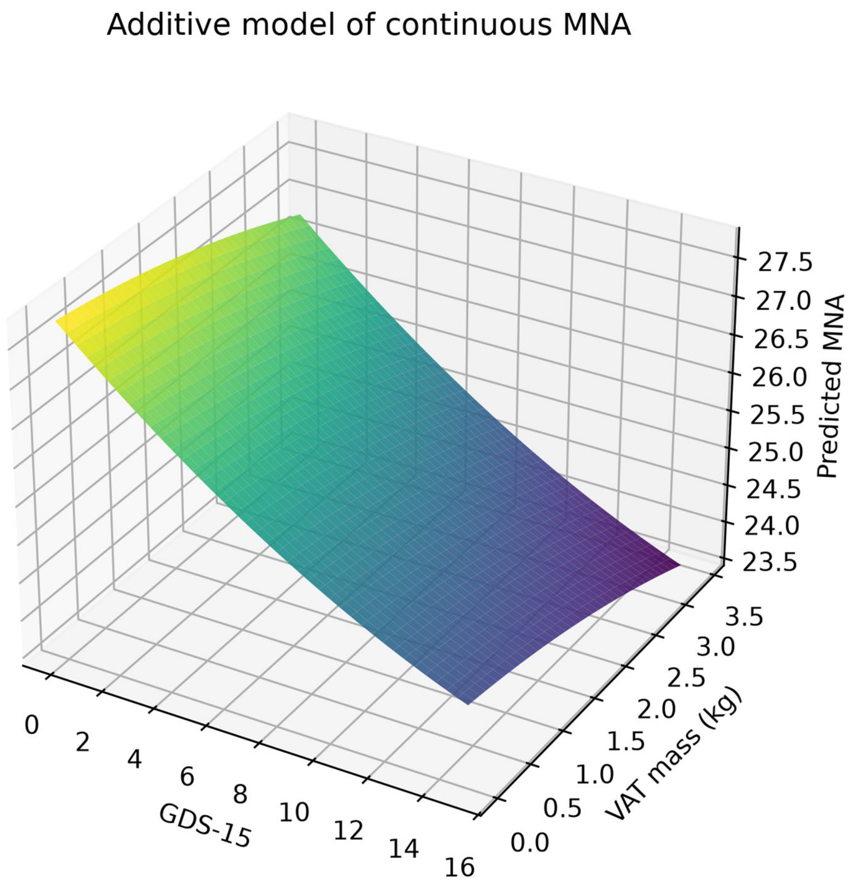

Supplementary Figure S1. *Exploratory response surface of the predicted continuous MNA score according to GDS-15 and VAT mass. The surface was estimated from an additive model including linear and quadratic terms for GDS-15 and VAT among participants with MNA scores at or below the instrument maximum of 30. The separately tested GDS-by-VAT interaction was non-significant (p = 0.72); the surface is therefore descriptive and should not be interpreted as evidence of interaction or causality. GDS, Geriatric Depression Scale; MNA, Mini Nutritional Assessment; VAT, visceral adipose tissue.*

Supplementary Figure S2

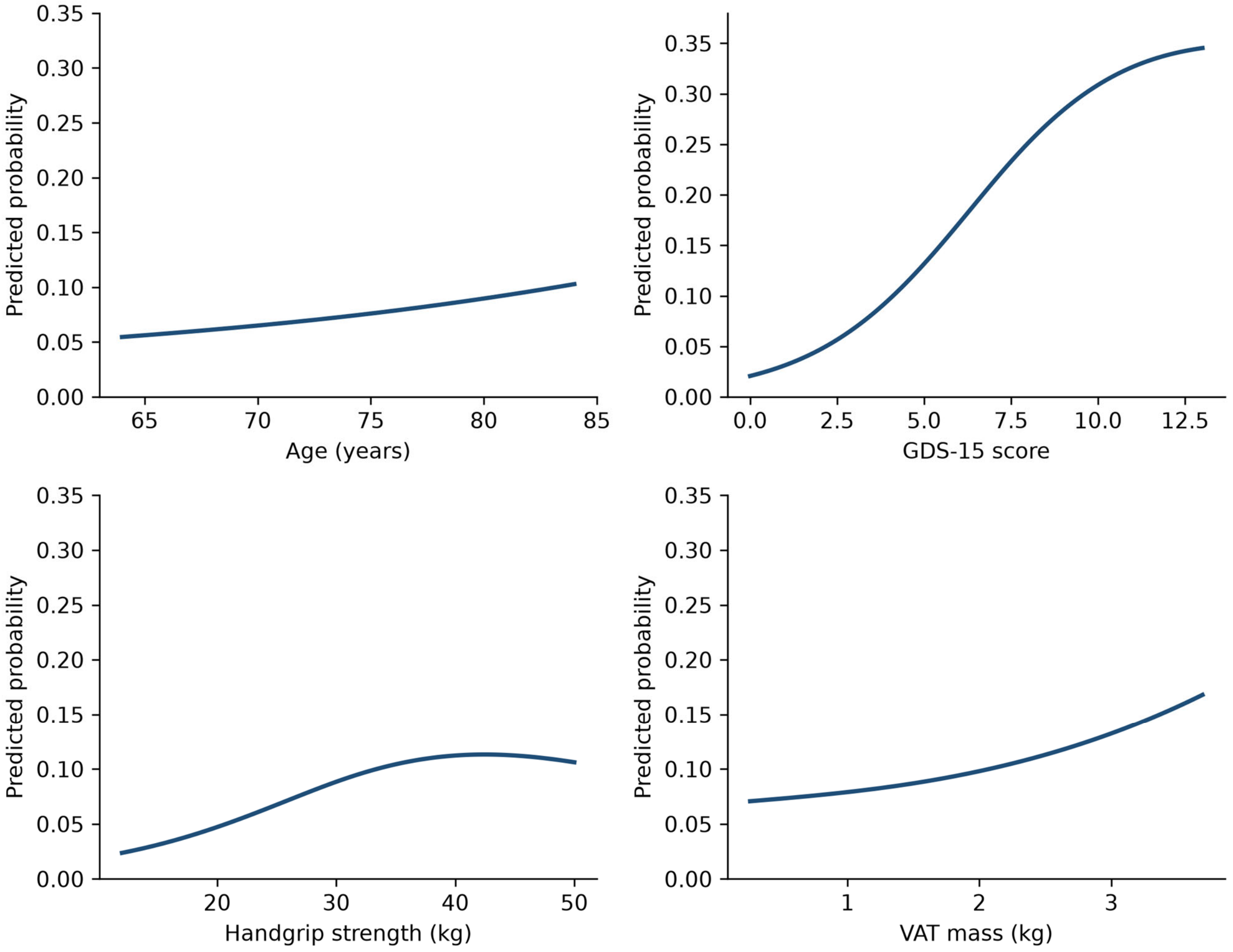

Supplementary Figure S2. *Adjusted flexible-form associations of age, GDS-15, handgrip strength and VAT mass with the predicted probability of MNA-defined nutritional risk. Curves were obtained from logistic models using natural cubic splines with three degrees of freedom. Predictions for age, GDS-15 and handgrip were based on the primary model, while the VAT curve came from the age-, sex- and GDS-adjusted VAT model; other covariates were held at representative values. The curves are descriptive, and formal tests for departure from linearity are reported in Supplementary Table S5. GDS, Geriatric Depression Scale; MNA, Mini Nutritional Assessment; VAT, visceral adipose tissue.*
